# Diagnosis and Treatment of Invasive Aspergillosis Caused by Non-*fumigatus Aspergillus* spp.

**DOI:** 10.3390/jof9040500

**Published:** 2023-04-21

**Authors:** Jannik Stemler, Christina Többen, Cornelia Lass-Flörl, Jörg Steinmann, Katharina Ackermann, Peter-Michael Rath, Michaela Simon, Oliver Andreas Cornely, Philipp Koehler

**Affiliations:** 1 Department I of Internal Medicine, Center for Integrated Oncology Aachen Bonn Cologne Duesseldorf (CIO ABCD), European Diamond Excellence Center for Medical Mycology (ECMM), Faculty of Medicine, University Hospital of Cologne, University of Cologne, 50937 Cologne, Germany; 2 Institute of Translational Research, Cologne Excellence Cluster on Cellular Stress Responses in Aging-Associated Diseases (CECAD), University of Cologne, 50923 Cologne, Germany; 3 German Centre for Infection Research (DZIF), Partner Site Bonn-Cologne, 50923 Cologne, Germany; 4 Institute of Hygiene and Medical Microbiology, European Diamond Excellence Center for Medical Mycology (ECMM), Medical University of Innsbruck, 6020 Innsbruck, Austria; 5 Institute of Clinical Hygiene, Medical Microbiology and Infectiology, Paracelsus Medical University, Klinikum Nürnberg, 90419 Nuremberg, Germany; 6 Institute of Medical Microbiology, University Hospital Essen, European Diamond Excellence Center for Medical Mycology (ECMM), 45147 Essen, Germany; 7 Institute for Medical Microbiology, Immunology and Hygiene, Faculty of Medicine, University Hospital of Cologne, University of Cologne, 50937 Cologne, Germany; 8 Clinical Trials Centre Cologne (ZKS Köln), University of Cologne, 50935 Cologne, Germany

**Keywords:** invasive aspergillosis, epidemiology, invasive fungal disease, immunocompromised host, surgery, antifungal treatment, antifungal stewardship

## Abstract

With increasing frequency, clinical and laboratory-based mycologists are consulted on invasive fungal diseases caused by rare fungal species. This review aims to give an overview of the management of invasive aspergillosis (IA) caused by non-*fumigatus Aspergillus* spp.—namely *A. flavus*, *A. terreus*, *A. niger* and *A. nidulans*—including diagnostic and therapeutic differences and similarities to *A. fumigatus*. *A. flavus* is the second most common *Aspergillus* spp. isolated in patients with IA and the predominant species in subtropical regions. Treatment is complicated by its intrinsic resistance against amphotericin B (AmB) and high minimum inhibitory concentrations (MIC) for voriconazole. *A. nidulans* has been frequently isolated in patients with long-term immunosuppression, mostly in patients with primary immunodeficiencies such as chronic granulomatous disease. It has been reported to disseminate more often than other *Aspergillus* spp. Innate resistance against AmB has been suggested but not yet proven, while MICs seem to be elevated. *A. niger* is more frequently reported in less severe infections such as otomycosis. Triazoles exhibit varying MICs and are therefore not strictly recommended as first-line treatment for IA caused by *A. niger*, while patient outcome seems to be more favorable when compared to IA due to other *Aspergillus* species. *A. terreus*-related infections have been reported increasingly as the cause of acute and chronic aspergillosis. A recent prospective international multicenter surveillance study showed Spain, Austria, and Israel to be the countries with the highest density of *A. terreus* species complex isolates collected. This species complex seems to cause dissemination more often and is intrinsically resistant to AmB. Non-*fumigatus* aspergillosis is difficult to manage due to complex patient histories, varying infection sites and potential intrinsic resistances to antifungals. Future investigational efforts should aim at amplifying the knowledge on specific diagnostic measures and their on-site availability, as well as defining optimal treatment strategies and outcomes of non-*fumigatus* aspergillosis.

## 1. Introduction

Increasing drug resistance and rising numbers in incidence and mortality due to invasive fungal disease (IFD) are worrisome trends in medical mycology [1,2,3,4].

Molds cause a broad spectrum of diseases, while the development of IFD generally depends on the underlying immune status and other host factors. Invasive aspergillosis (IA) is an IFD of major importance among severely immunocompromised patients, especially those with long-term neutropenia, acute leukemia, glucocorticoids or other immunosuppressive drugs, in recipients of hematopoietic stem cell transplantation (HSCT), or solid organ transplantation (SOT) or in patients with inherited immune disorders [5,6,7,8].

The genus *Aspergillus* comprises more than 350 accepted species in the literature to date, of which at least 60 species were identified to cause invasive infection in humans [9]. In patients with IA, *A. fumigatus* species complex remains the most commonly identified species, with a proportion of 46% to 89% of tested clinical isolates [8,9,10]. Due to the increased implementation of azole antifungal prophylaxis, antifungal use in agriculture and developments in diagnostic capacities, a changing epidemiology of *Aspergillus* spp. has been described [11].

Non-*fumigatus* IA cases are infrequently described, and infections are often not analyzed systematically in prospective studies, however, their detection seems to become more frequent in clinically relevant samples. Clinical management strategies are most of the time applied in analogy to IA caused by *A. fumigatus*. However, non-*fumigatus Aspergillus* spp. is a heterogenous group comprising several subgenera and sections [8]. Especially in tropical and subtropical regions, non-*fumigatus* species even seem to be the leading cause of IA [12]. Due to intrinsic resistance patterns, genus identification is mandatory for clinical management. This review addresses the specifics of the diagnostic and therapeutic management of non-*fumigatus* IA.

## 2. Methods

**Figure 1:**
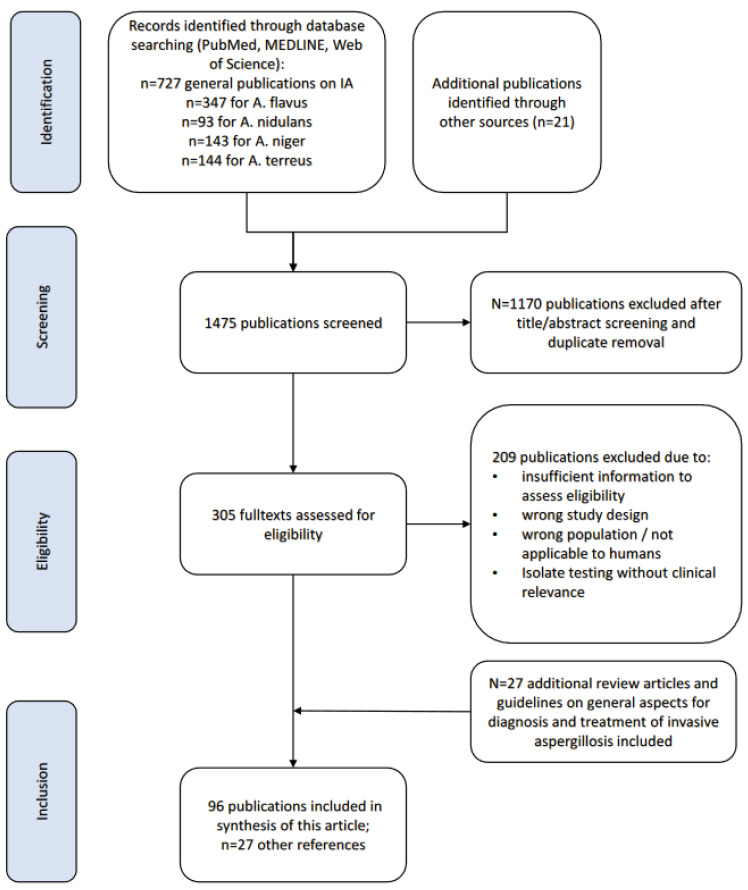
Selection process of publications.

We performed a PubMed search-based literature review on non-*fumigatus* IA cases. The search was restricted to English language articles from database inception until 2023. Search terms were selected focusing on IA specifically caused by the non-*fumigatus Aspergillus* spp. *A. flavus*, *A. terreus*, *A. niger* and *A. nidulans* (Supplementary Table S1). Publications were selected based on title and abstract. Studies on aspergillosis, in general, were included for epidemiological purposes if isolates and/or infections of the respective non-*fumigatus Aspergillus* spp. were investigated. Guideline recommendations, book chapters and well-designed reviews were considered to be included in elaborations on general aspects regarding diagnostics and treatment. All included publications were checked for their primary literature and included if additional references became available (Figure 1).

## 3. Results

### General Aspects

The general diagnostic approach to IA is complex and requires awareness and involvement of multiple medical disciplines as well as a broad portfolio of diagnostic measures. Patient populations affected by IA are immunosuppressed, as described above. Furthermore, patients in need of intensive care for viral pneumonia, in particular influenza-associated pulmonary aspergillosis (IAPA) or COVID-19-associated pulmonary aspergillosis (CAPA), trauma or post-surgery patients, or inherited or acquired immunodeficiencies, for example, chronic granulomatous disease (CGD) [13,14]. Of note, disseminated aspergillosis has been described more frequently for IA due to non-fumigatus spp. than for *A. fumigatus*, especially in the hematological population.

For some high-risk patient populations, specifically those with acute myeloid leukemia (AML) and allogeneic HSCT recipients, antifungal prophylaxis has been shown to reduce IFD incidence and improve overall survival [15,16].

Based on the underlying disease, chest X-ray or preferably computerized tomography (CT) can reveal fungal masses (aspergilloma) in chronic pulmonary aspergillosis or characteristic signs of IA [17]. In neutropenic patients, radiological signs suggestive of invasive pulmonary aspergillosis (IPA) are nodules with a surrounding ground-glass opacity (“halo sign”) indicating micro-hemorrhage and vessel infiltration as well as the air-crescent sign, which occurs when infiltrates connect to air-spaces late in the natural course of IA or during successful antifungal treatment [18]. In certain high-risk groups, such as patients with AML, it can be beneficial to screen as early as upon hematologic malignancy diagnosis for early manifestations of IPA by “baseline” chest CT [19,20,21]. Other infected sites should be examined by CT, positron-emission tomography (PET)-CT or magnet resonance imaging (MRI) to identify disseminated disease and rule out bone destruction, abscess formation or blood vessel infiltration [22].

Radiological examinations may support the suspicion of IFD but cannot help to identify the causing fungal pathogen and species identification. Subsequent steps involve obtaining adequate specimens for microscopy and fungal staining, fungal culture, the biomarkers galactomannan (GM) enzyme immunoassay (EIA) and 1,3-ß-D-glucan (BDG), and targeted polymerase chain reaction (PCR) assays [23,24].

Serum GM EIA is a rapid and non-invasive, and helpful tool if applied in the appropriate patient cohort [25]. Sensitivity of serum and bronchoalveolar lavage (BAL) GM is best validated for neutropenic patients, while BAL GM has a higher sensitivity than serum GM [23]. In neutropenic patients, the combination of two serological assays (GM EIA and BDG) improves the specificity and positive predictive value of each individual test (up to 100%) [26,27].

While microscopy and biomarkers are less specific, culture has the advantage of identifying the specific etiological agent and allowing antifungal susceptibility testing [17]. Culture on Sabouraud dextrose agar, malt extract agar or specific other culture media remains the method of choice in order to perform morphologic and microscopic diagnosis of the specific species, as well as a starting point for antifungal susceptibility testing. The primary culture should be incubated for at least 10 days [28]. For phenotypic identification, cultivation on Petri dishes is recommended. To define the temperature tolerance, strains are precultured on slants and subsequently transferred to Petri dishes and incubated at 25, 35 to 40 and 42 °C [28]. For microscopy, culture material is taken with a sellotape, put on a slide and stained with lactophenol blue, and afterward examined by light microscopy. The microscopic examination of sexually and asexually produced structures is used for species identification. [29].

Culture and cytology in BAL have high specificity but low sensitivity in neutropenic patients [23]. Conventional nested *Aspergillus* PCR in BAL in immunocompromised patients is more and more frequently applied but also has limited sensitivity in patients with a low probability of IFD and without high fungal burden [30]. PCR is frequently used with a variety of commercially available and in-house kits targeting pan-fungal markers. Common genetic targets are ITS regions, calmodulin, beta-tubulin and actin genes, among others [31]. It is important to underline that the performance of these techniques depends on the availability and quality of the databases employed [31]. PCR-ELISA may improve the rates of early diagnosis of IA, and the combination of PCR-ELISA and GM assay increased sensitivity to 83% and negative predictive value to 98% [32].

Another diagnostic tool is matrix-assisted laser desorption ionization time-of-flight (MALDI-TOF) mass spectrometry [33]. However, it is not frequently used for species identification in IA due to the complex methodology for molds, the lack of in-house databases for species identification and the heterogeneity of spectra within *Aspergillus* species [34].

In vitro, antifungal susceptibility testing is usually performed against available antifungals using the European Committee on Antimicrobial Susceptibility Testing (EUCAST) or Clinical and Laboratory Standards Institute (CLSI) broth microdilution method or gradient strip test [35]. For *Aspergillus* spp. other than *A. fumigatus*, a clinical breakpoint according to the EUCAST methodology for most antifungals has not yet been defined due to a lack of data. However, for the most frequently used triazole antifungals and polyenes, epidemiological cut-off values (ECOFFs) are available for discrimination of wild type isolates from isolates with acquired resistance [36] (Table 1).

Definitive diagnosis of a proven IA requires evidence of fungi in direct microscopy of a normally sterile specimen or histopathology using fungal staining such as Gomori-Grocott staining showing tissue invasion with septate acute angle branching hyaline hyphae [37,38].

Cryptic species are those that are morphologically indistinguishable from others but differentiated by molecular methods. Clinically important cryptic species include *A. alliaceus*, *A. calidoustus*, *A. felis*, *A. lentulus*, *A. tubingensis*, *A. viridinutans* and *Neosartorya pseudofischeri*. In these species, intrinsic antifungal resistance has often been noted, but knowledge of susceptibility patterns, MIC breakpoints, and response to antifungal therapy is limited [31].

Treatment of IA due to non-*fumigatus Aspergillus* spp. involves antifungals, but needs evaluation of surgical options, too [39,40,41,42]. Broad-spectrum triazoles isavuconazole, posaconazole or voriconazole are the drugs of choice for IA [43,44,45]. For isavuconazole, the 2017 ESCMID guideline only marginally recommends its use for the treatment of IA due to *A. niger*, but data seems to be contradicting as this drug was effective during treatment for non-fumigatus IA in another study. The polyene amphotericin B (AmB), especially in its liposomal formulation, is licensed for empiric antifungal treatment in persistent febrile neutropenia as well as a targeted treatment for IA [46,47]. However, for some non-*fumigatus Aspergillus* spp. discussed here, particularly high MICs for AmB have been described. Echinocandins inhibit the growth of *Aspergillus* spp. in vivo and are available as treatment alternatives when triazoles and AmB are not available or contraindicated due to toxicity [48]. However, there are no randomized controlled trials for their use as single agents in IA, and they should not be used for IA with involvement of the central nervous system (CNS) due to limited penetration [42]. Combination treatment consisting of an azole plus an echinocandin may be implemented for severe or refractory cases with improved outcomes compared to azole monotherapy [49,50]. A relation between the MIC and the clinical outcome has been described for *A. flavus* and *A. terreus* infections, but not for *A. fumigatus* [51,52,53,54,55]. Novel antifungals for the treatment of IA, such as fosmanogepix, ibrexafungerp, olorofim or opelconazole are currently under investigation in clinical trials and may be promising options for azole-resistant IA, oral outpatient treatment, patients with toxicity or drug-drug interactions (DDI) during treatment with currently available antifungals, and in refractory cases [56].

**Table 1:** Overview of characteristics of *A. fumigatus* compared to *A. flavus*, *A. nidulans*, *A. niger* and *A. terreus*.

	*A. fumigatus*	*A. flavus*	*A. nidulans*	*A. niger*	*A. terreus*
Phylogeny—Subgenus and Section	*Fumigati* *Fumigati*	*Circumdati* *Flavi*	*Nidulantes* *Nidulantes*	*Circumdati* *Nigri*	*Circumdati* *Terrei*
Environmental sources	Ubiquitous in the environment, construction work, dust, soil, compost	Ubiquitous in the environment; also identified in compost	Ubiquitous in the environment	Ubiquitous in the environment	Ubiquitous in the environment; compost, soil, construction work, dust
Epidemiology	Worldwide identification	Higher incidence in warm, arid climate zones (tropical and subtropical climate zones)	Worldwide identification	Worldwide identification	More often identified in Austria, Israel, Spain and Texas, USA
Morphology					
- Colony color	- Blue-green	- Yellow-green	- Green, cream-buff or honey-yellow	- Black	- Yellow-brown to cinnamon-brown
- Conidial heads	- Columnar	- Radiate	- Columnar	- Radiate	- Densely columnar
- Conidiogenous cells	- Uniseriate	- Uni- and biseriate	- Biseriate	- Biseriate, metulae twice as long as phialides	- Biseriate, metulae and phialides of equal length
- Conidia	- Verrucose	- Echinulate	- Green in mass	- Brown, subspherical	- Spherical to ellipsoidal
- Conidiophore	- Smooth-walled	- Rough-walled	- Brownish stipes	- Smooth-walled	- Smooth walled
Microbiological test peculiarities	Microscopy, culture, PCR, Galactomannan in BAL or serum; Hypersensitivity tests for ABPA	Microscopy, culture, PCR, Galactomannan in BAL or serum (reduced sensitivity)	Microscopy, culture, PCR, Galactomannan in serum or BAL;	Microscopy, culture, PCR, Galactomannan in serum or BAL;	Microscopy, culture, PCR, Galactomannan in serum or BAL;
Disease entities	IPA, sinusitis and cerebral IA; chronic aspergillosis including aspergilloma; allergic bronchopulmonary aspergillosis	IPA, sinusitis, otomycosis, cutaneous IA; osteomyelitis; rarely aflatoxin-induced diseases	IPA, osteomyelitis	IPA, superficial infections in chronically ill patients: otomycosis, sinusitis	IPA, Higher rate of disseminated disease reported
Main risk factors for IFI	Long-term neutropenia (acute leukemias, HSCT), GvHD, SOT, ICU patients with viral pneumonia (CAPA, IAPA)	Long-term neutropenia (acute leukemias, HSCT), SOT, Trauma, diabetes	Neutropenia, CGD	Long-term neutropenia (acute leukemias, HSCT), SOT; frequently identified colonizer in immunocompetent individuals	Neutropenia, HSCT, liver transplant
Treatment until susceptibility test results available	Triazoles (isavuconazole, posaconazole, voriconazole), (liposomal) AmB	Triazoles (isavuconazole, posaconazole, voriconazole), consider combination therapy	Voriconazole	Avoid azole monotherapy; consider combination with (liposomal) AmB	Triazoles (isavuconazole, posaconazole, voriconazole)
Peculiarities in treatment (according to MIC)	Azole resistance in Europe increasing	Sometimes reduced susceptibility for AmB (≥4 mg/L); if MIC ≥2 mg/L combination treatment with echinocandins suggested	Reduced susceptibility for AmB (published MICs between ≥1 mg and 4 mg/mL), innate resistance to AmB suggested	Commonly reported elevated MICs of azoles compared to A. fumigatus (2 µg/mL, 1 µg/mL, 4 µg/mL, and 1 µg/mL for AmB, isavuconazole, itraconazole, and voriconazole, respectively), lower MIC for posaconazole (0.5 µg/mL) and caspofungin (0.25 µg/mL) (CLSI) [57]; if available use other azoles than itraconazole or isavuconazole	Avoid AmB (most species with intrinsic resistance and MIC >2 mg/L)

Abbreviations: AML, acute myeloid leukemia; BAL, bronchoalveolar lavage; CAPA, COVID-19 associated pulmonary aspergillosis; CGD, chronic granulomatous disease; CLSI, clinical and laboratory and standards institute; GvHD, graft-versus-host disease; HSCT, hematopoietic stem cell transplantation; IA, invasive aspergillosis; IAPA, influenza-associated pulmonary aspergillosis; IPA, invasive pulmonary aspergillosis; ICU, intensive care unit; MIC, minimum inhibitory concentration; PCR, polymerase chain reaction; SOT, solid organ transplantation.

## 4. *A. flavus*

### 4.1. General Characteristics and Epidemiology

*Aspergillus flavus* is the second most frequently isolated *Aspergillus* spp. from clinical samples [58,59,60]. There seems to be a higher incidence of IA caused by *A. flavus* in warm and arid climate zones due to its ability to withstand higher temperatures [38,61]. Therefore, most reports of invasive infections are from sub-tropical countries like India, Pakistan, Saudi Arabia, Tunisia or Mexico (Table 1) [62].

IA due to *A. flavus* usually manifests as IPA but causes bronchopulmonary infections less frequently compared to *A. fumigatus*. Furthermore, it is the most frequently detected fungal pathogen of sinu-orbital and cerebral IFD as well as a frequent causative agent of invasive sinusitis and otitis externa in immunosuppressed patients. It also occurs in cutaneous IA and fungal keratitis, with the highest risk in patients after ocular surgery and diabetes [38,63]. Risk factors for the development of primary osteomyelitis due to *A. flavus* are trauma and diabetes [38,64]. In a review of bone and joint aspergillosis, *A. flavus* was the causative agent in 18% of patients, including the manifestations of mastoiditis, discitis, vertebral osteomyelitis and septic arthritis [65]. Arthritis and/or osteomyelitis usually develop as a secondary infection in disseminated aspergillosis after pulmonary aspergillosis or endocarditis with hematogenous spread [38]. Cardiac aspergillosis due to *A. flavus* has been described rarely and may present as endocarditis or after cardiac surgery and is mainly a healthcare-acquired infection [66,67].

### 4.2. Diagnosis and Microbiology

**Figure 2:**
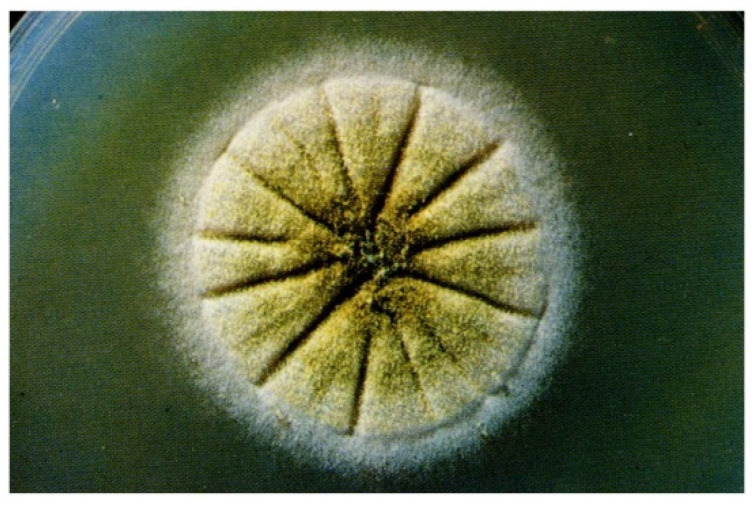
*Aspergillus flavus* macromorphology on SDA, incubation for 6 days at 30 °C.

*Aspergillus flavus* grows well on Sabouraud dextrose agar or Czapek Dox and malt extract at 37 °C, and germination of conidia occurs at about 24 h (Figure 2) [38].

The key microscopic finding for *A. flavus* is biseriate conidial heads with a yellow to green or brown appearance and dark sclerotia [60]. Besides the general diagnostic approach for IA and conventional microscopic approaches, immunohistochemistry with in situ hybridization with WF-AF-1 or EB-A1 antibodies specific for *A. flavus* (as well as *A. fumigatus* and *A. niger*) may be of use. However, this technique is time-consuming and not available in many centers [68,69].

For serologic testing, the sensitivity of GM was shown to be lower for *A. flavus* when compared to *A. fumigatus* [70]. A combination of an *A. flavus*-targeted PCR-ELISA and GM testing showed an improved rate of early IA diagnosis in patients with hematological malignancies [32]. This may specifically be applied to patients without antifungal prophylaxis.

Susceptibility testing should be performed on all clinically relevant *A. flavus* samples to focus on reported elevated MICs for AmB. *A. flavus* has shown broadly variable MICs with shares between 66.6% and 92% of isolates being higher than ≥2 mg/L for AmB. depending on study and region suggestive of intrinsic resistance [71,72]. In addition, azole resistance seems to play a role in India, with 2.5% to 5% of isolates tested being resistant to commonly used triazoles [73,74]. Resistance mechanisms superior to those described for IA, in general, have been detected for *A. flavus*, such as mutations in the *cyp51a*, *cyp51b* and *cyp51c* alleles as multiple mechanisms responsible for azole resistance [74,75].

### 4.3. Clinical Management and Treatment

The recommended first-line antifungal treatment agent is voriconazole. Posaconazole and isavuconazole are reasonable alternatives with better tolerability [42,44,72]. Posaconazole has been noted to inhibit the lanosterol 14α-demethylase to a larger extent than other azoles in vitro [38]. In cases with a MIC ≥ 2 mg/L for voriconazole, combination therapy with caspofungin has been suggested to improve overall survival in modeling studies [76], as all echinocandins seem to demonstrate good activity against *A. flavus* in vitro [72].

The proposed ECOFF for AmB by EUCAST ranges at 4 mg/L. Therefore, if available, this may be used to guide therapy in patients with IA due to *A. flavus*. A correlation between the MIC and the clinical outcome of *A. flavus* infections has been described. Thus, L-AmB should be avoided as first-line treatment (Table 1) [31,71].

In patients with fungal sinusitis, fungal endophthalmitis and fungal otitis, bone erosion and subsequent spread to the CNS may occur [22,38]. For these sites of infection, local treatment (e.g., intracameral and intravitreal application) with antifungals is recommended as an addition to systemic therapy [74,77]. In these cases, surgical debridement should be performed to improve outcomes [22].

## 5. *A. nidulans*

### 5.1. General Characteristics and Epidemiology

In patients with primary immunodeficiencies, *A. nidulans* is the second most common pathogen causing IA after *A. fumigatus* [78]. Among them, patients with CGD are to be mentioned as particularly susceptible to IFD due to disruption of the NADPH oxidase complex and subsequent lack of neutrophil extracellular trap formation [78,79].

Generally, *A. nidulans* is the third most common fungal pathogen causing osteomyelitis, and besides CGD, risk factors include hematological malignancies, SOT, diabetes, and chronic pulmonary diseases, among others [64]. Lungs are the primary site of infection, but less frequently, skin, lymph nodes and liver may be affected [80]. Furthermore, osteomyelitis due to *A. nidulans* occurs more often compared to *A. fumigatus* and more frequently involves small bones with substantially higher mortality [65,81]. *A. nidulans* infections seem to have a more aggressive course and cause disseminated disease more often [82].

IFDs are the most common cause of death in patients with CGD and are also caused by rare and resistant molds, which may partially be due to the broad and long-term use of antifungals [83,84]. For this population, therefore, systemic triazole-based prophylaxis is generally recommended [85,86]. The fact that *A. nidulans* is rarely found as a causative agent of IFD in other immunocompromised patients than CGD favors the hypothesis of a unique pathogen-host interaction and role of the NADPH-oxidase for innate host-defense, which still needs to be determined [87].

### 5.2. Diagnosis and Microbiology

**Figure 3:**
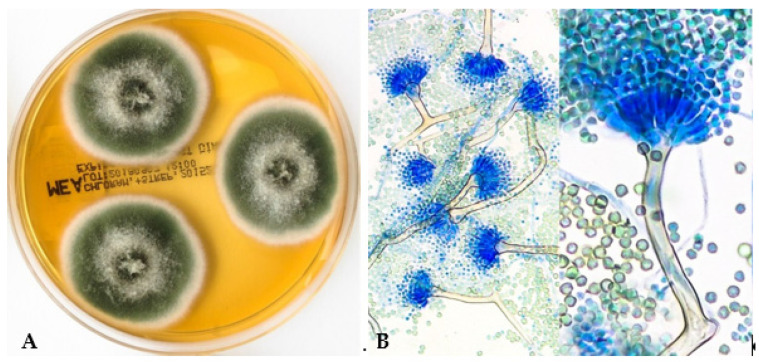
*Aspergillus nidulans* macro- and micromorphology. (**A**) *A. nidulans* on malt extract agar, incubation for 5 days at 35 °C. (**B**) Lactophenol cotton blue preparation (at ×400), columnar and biseriate conidial heads, brownish condiophores; Hülle cells are usually observed.

*Aspergillus nidulans* (also referred to as *Emericella nidulans* in its teleomorph form) shows rapidly growing, (dark) green, cream-buff or honey-yellow/orange colonies and reverse dark purplish appearance in culture (Figure 3A). Microscopically the conidial heads of *A. nidulans* are short, septated and columnar; the vesicles hemispherical, and the conidiogenous cells biseriate (Figure 3B). MALDI-TOF MS analysis may be used to identify *A. nidulans* in clinical samples [88]. GM is detectable in serum in patients with IA due to *A. nidulans*, but is less sensitive [89].

To select antifungal treatment, susceptibility testing is mandatory. For AmB, the MIC was >1 mg/L and up to 4 mg/L in some cases, using the EUCAST method [90]. The lowest MICs were noted for anidulafungin, micafungin, and posaconazole (<1 mg/L). Isavuconazole seems to have good in vitro activity with rather low proposed ECOFFs for *A. nidulans* as well [91]. In a study with patients with primary immunodeficiencies, *A. nidulans* isolates had low MICs for itraconazole, posaconazole and voriconazole but were elevated for AmB [78]. Thus, innate resistance of *A. nidulans* against AmB has been suggested [92].

### 5.3. Clinical Management and Treatment

The 2018 ESCMID guideline on the management of *Aspergillus* infections marginally recommends therapy with voriconazole with a low level of evidence, mostly due to the lack of studies involving enough patients with IA due to *A. nidulans* [42]. No primary recommendation was made for AmB, especially for patients with CGD, due to high MICs and documented poor clinical outcomes [82].

In cases with fungal osteomyelitis, the treating team needs to strive for surgical resection alongside a broad systemic antifungal therapy to achieve a higher rate of positive outcomes [64,79]. In those cases, voriconazole should be used for treatment as it provides high oral bioavailability and good penetration into bone tissue [65]. Though rarely performed, allogeneic HSCT represents a therapeutic option for patients with CGD with recurrent and refractory IFD [79]. The novel fungicidal agent olorofim seems to exhibit reasonable activity against *A. nidulans* in a mouse model [93]. In summary, treatment for IA due to *A. nidulans* does not differ substantially from general treatment approaches with close response monitoring when AmB is selected as upfront monotherapy.

## 6. *A. niger*

### 6.1. General Characteristics and Epidemiology

The cumulative 1-year incidence of infections with *A. niger* complex in patients with SOT or HSCT was 0.048% in a multicenter cohort study [7]. It is a rarely detected species in IA of post-transplant patients [50,94]. There seems to be an increase in the incidence of IA due to *A. niger* spp. complex from a mean incidence of 0.023 per 10,000 patient days to 0.095 per 10,000 patient days between 2005 and 2011 [95]. Within the *A. niger* spp. complex, *A. tubingensis* was most frequently isolated [95]. A possible explanation for the selection of triazole-resistant *Aspergilli tubingensis* could be the use of fungicides in agriculture or azole-based prophylaxis in certain patient populations [96]. *A. niger* has been reported to be a spore contaminant in burn wards which elucidates the role of hygiene measures for the prevention of IA, including filtered ventilation and decontamination of surfaces and personal equipment [97].

*Aspergilli* of the section *Nigri* have been identified as colonizers of the nose and throat in immunocompetent patients with other predisposing conditions, such as chronic lung diseases [98]. In this population, *A. niger* was also the most frequently detected pathogen causing otomycosis [99]. In a series of eight proven/probable *A. niger* cases in patients with hematological malignancies, three of which were breakthrough IA during systemic antifungal prophylaxis, the infected sites were the lungs and paranasal sinuses. Patients with AML were particularly at risk, and most acquired the fungal infection during remission-induction chemotherapy. IA-related mortality was as high as 75% [100]. Furthermore, *A. niger* is the second most common *Aspergillus* spp. found in the setting of fungal peritonitis in peritoneal dialysis patients [101].

Overall, larger epidemiological studies have shown a higher survival rate in patients with IA due to *A. niger*—about 80% vs. 66% and 60.5% compared to *A. fumigatus* and *A. flavus*, respectively [8].

### 6.2. Diagnosis and Microbiology

**Figure 4:**
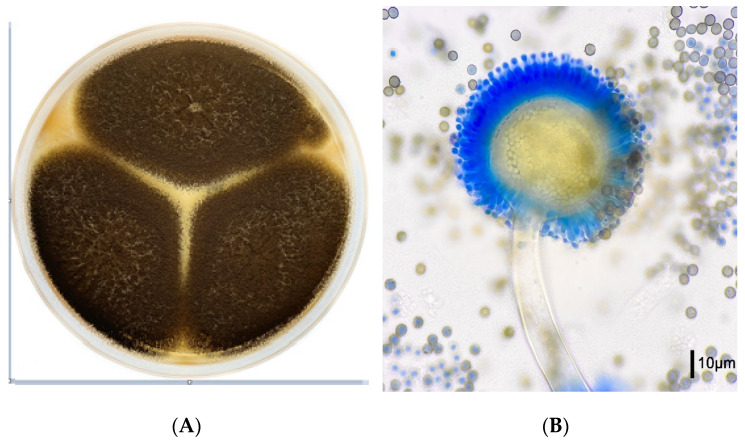
*Aspergillus niger* macro- and micromorphology. (**A**) *A. niger* on malt extract agar, incubation for 4 days at 35 °C. (**B**) Lactophenol cotton blue preparation (at ×400), radiate and biserate conidial heads.

*Aspergilli* of the section *Nigri* grow on all mycological media within one to two days, as they do not need special culture conditions. *A. niger* shows black colonies in culture (Figure 4A). Microscopically the conidial heads radiate, the vesicles subspherical and the conidiogenous cells biseriate (Figure 4B).

The species within the section are morphologically indistinguishable, but identification at the species level is usually not necessary [102]. If required, identification of *A. niger* can also be performed with the MALDI-TOF MS analysis [88]. Where *Aspergillus* isolates cannot be clearly identified by conventional phenotypic methods, DNA sequencing is often helpful [103].

There are no clinical data on the value of GM or (1-3)-ß-D-glucan in serum for diagnosis of invasive infection, but GM is detectable in serum in patients with IA due to *A. niger* [89]. Specific PCR-based assays to detect *A. niger* DNA in clinical specimens are commercially not available.

In general, *Aspergilli* of the section *Nigri* are susceptible to all antifungal drugs, and breakpoints for *A. niger* have been proposed by EUCAST [104]. However, data on antifungal susceptibility testing of *A. niger* isolates seem to be contradictory. CLSI methodology yielded epidemiological cutoff values of 2µg/mL, 1µg/mL, 4µg/mL and 1µg/mL for AmB, and isavuconazole, itraconazole and voriconazole, respectively, and was the lowest for caspofungin and posaconazole (both 0.25 µg/mL) [105]. Breakthrough IFDs have also been observed under voriconazole therapy, which is why the use of voriconazole has been rather discouraged [95]. Among the *A. niger* spp. complex, *A. tubingensis* shows particularly high MICs to azoles, especially to itraconazole [95,106,107]. It was shown that *A. niger* isolates were 100% resistant to ketoconazole and 33% resistant to AmB and itraconazole but showed no resistance to caspofungin [108]. In summary, MIC values for *A. niger* were generally higher than those for *A. fumigatus*; whether this translates into a poorer clinical response is unknown [109].

### 6.3. Clinical Management and Treatment

Compared to *A. fumigatus*, infections due to *A. niger* appear to be less virulent and are less frequently associated with invasiveness, as they are up to 10 times more frequently reported in superficial mycoses [94,110]. Nonetheless, invasive *A. niger* infections remain difficult to treat due to varying susceptibility patterns. Guidelines recommend avoiding azole monotherapy due to higher MICs and moderately recommend against the use of itraconazole and isavuconazole for the same reason, however, with a low level of clinical evidence [42,111]. First-line AmB treatment in hematological patients showed a response in only 25% of cases. However, with a very low number (*n* = 8) of patients treated for IA due to *A. niger* [100]. In cases of peritoneal IA in peritoneal dialysis patients, removal of the indwelling catheter and intra-peritoneal antifungals improves outcome [101]. With the lung being the most frequently affected site in invasive *A. niger* infections, surgical approaches may hardly be feasible. Overall mortality seems to be higher in patients with hematological malignancies and in the ICU compared to those with SOT and chronic pulmonary diseases [95].

## 7. *A. terreus*

### 7.1. General Characteristics and Epidemiology

Within the genus *Aspergillus*, *A. terreus* species complex takes a special position, as most representatives are AmB resistant, and invasive infections are frequently associated with the dissemination and poor outcome [112].

*A. terreus* accounts for four to 12% of clinically relevant *Aspergillus* isolates [113,114]. It is described as the most important species within the section *Terrei* and is found commonly in soil, dust, compost and other environmental sources [115]. It appears that *A. terreus* is a fungal pathogen of increasing importance in patients with IA. In an international multicenter study, the prevalence of *A. terreus* was 5.2% among all reported aspergillosis cases [114].

Generally, patients with hematological malignancies, especially acute leukemia and long-term neutropenia, are at risk of developing *A. terreus* IA. Reporting or observer bias may play a role in the description of the epidemiology of such rare fungal species depending on the local research efforts and the variable contribution to international studies in the field.

### 7.2. Diagnosis and Microbiology

Molecular and phylogenetic studies divide the *Aspergillus* section *Terrei* into a total of 17 accepted species distributed over three series (*Ambigui*, *Nivei*, *Terrei*) [112]. Only micromorphology or targeted molecular-based analyses support discrimination of this pathogen from other *Aspergillus* species, such as from the sections *Fumigati, Flavi* and *Nigri* [17].

**Figure 5:**
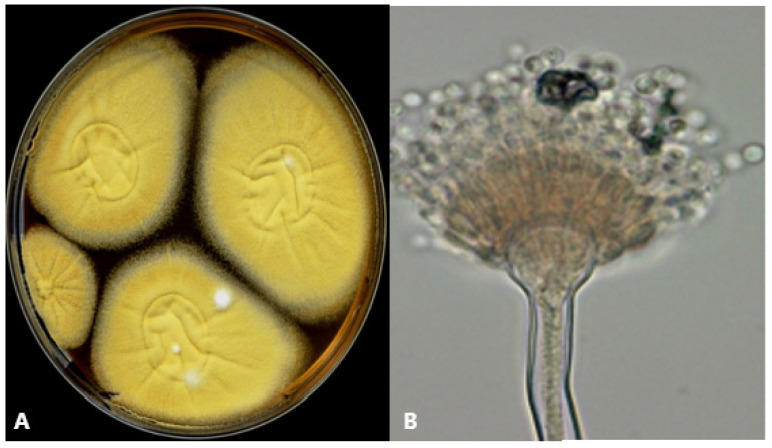
*Aspergillus terreus* macro- and micromorphology. (**A**) A. *terreus* on malt extract agar, incubation for 4 days at 35 °C. (**B**) Microscopy (at ×400), radiate and biserate conidial heads.

On Czapek or Sabouraud dextrose agar, *A. terreus* colonies grow from beige to buff to cinnamon and are able to become floccose, the reverse is yellow, and yellow soluble pigments are frequently present (Figure 5A). Conidial heads are compact, columnar (reaching up to 500 μm long by 30 to 50 μm in diameter), and biseriate; conidiophores are hyaline and smooth-walled; conidia are globose-shaped, smooth-walled, 1.5 to 2.5 μm in diameter, and vary in color from brown to light yellow (Figure 5B) [112]. Only *A. allahabadii* and *A. neoindicus* produce white conidia. The production of accessory conidia seems to be specific to *A. terreus* species complex, as described for *A. terreus* sensu strictu, *A. citrinoterreus*, *A. hortai*, *A. alabamensis* and *A. neoafricanicus* [112]. The formation of these non-pigmented aleurioconidia, which are settled directly from vegetative hyphae—reveal under both in vitro and in vivo conditions. Additionally, accessory conidia have been described for species of the *Aspergillus* section *Flavipedes*, which seem to form an evolutionary sister branch of the section *Terrei* [112].

### 7.3. Clinical Management and Treatment

The most relevant clinical feature of *A. terreus* is the intrinsic resistance to AmB. Therefore, identification of *A. terreus* at the species level is crucial to exclude AmB treatment. MIC testing should be performed while clinical breakpoints are available for isavuconazole, itraconazole and posaconazole.

First-line treatment should be based on triazoles which have shown better response rates and greater survival in hematology patients [116].

*A. terreus* infections seem to be associated with a poorer outcome than compared to *A. fumigatus* infections [116,117]. An almost twice as high rate of disseminated infections has been described in one single-center study, and higher mortality compared to IA caused by other species [116].

## 8. Discussion

There is a large variety of IFD caused by non-*fumigatus Aspergilli*. Their epidemiology is still not well described, and clinical evidence is scarce, of heterogenous quality and often outdated.

One older study reported a high incidence of non-*fumigatus* IA in neutropenic patients with similar IA-attributed mortality in the *A. fumigatus* and non-*fumigatus* groups [109]. Other studies suggested a comparable distribution of infectious sites and mortality independent of affected immunosuppressed subpopulation and involved *Aspergillus* spp. [8]. Furthermore, it appears that these rarely detected species are more often present in IA in patients with primary immunodeficiencies [78]. This suggests that fungal pathogenesis differs in this small patient population compared to patients with iatrogenic immunosuppression and creates an epidemiological niche for certain fungal pathogens.

To diagnose, general recommendations and procedures for collection, transport and storage of clinical specimens, direct microscopic examination, isolation and identification procedures are equally valid for all *Aspergillus* species. Whether or not the precise species identification may have an impact on individual clinical management remains to be determined. However, its value for epidemiological surveillance and outbreak investigation has been documented beyond doubt [118,119]. Molecular methods should be implemented and made available in routine practice for exact species identification to improve and accelerate the diagnosis of these IFDs. Unfortunately, these techniques rely on the availability of laboratory resources and databases and are not available in many centers, especially in low- and middle-income countries [120,121,122].

Susceptibility patterns suggest more differences to *A. fumigatus* than yet described [123]. Non-*fumigatus Aspergillus* spp. has been suspected to exhibit innate AmB resistance, which has been described for both *A. nidulans* and *A. terreus*, as well as elevated MICs for AmB for other strains [92]. However, this has not yet been fully proven in vivo, despite varying MICs for non-*fumigatus Aspergilli* in several studies. This elucidates the importance of identification by culture and antifungal susceptibility testing to guide antifungal therapy. The patient outcome has been correlated with in vitro MICs of AmB [71]; it has been documented for *A. flavus* and *A. niger* but not for *A. fumigatus* so far [53,54]. Whether in vitro susceptibility patterns generally translate into the clinical response is, therefore, not yet fully determined.

Further *Aspergillus* species than the ones presented in this review are identified in clinical samples and IFD with increasing frequency [124]. Efforts to perform clinical studies on IFD caused by one fungal species only are much appreciated and enrich the knowledge on differences between the different *Aspergillus* spp., a gap that may be closed by global registry studies [125].

The general diagnostic and therapeutic approach for suspected IA due to non-*fumigatus Aspergillus* spp. does not differ substantially from the work-up and measures taken for any IFD. Host factors, as outlined above, and local epidemiology should be considered. Diagnosis includes adequate sampling and examination via microscopy and culture-based procedures and, if available, molecular tests such as specific PCR for *A. flavus* to differentiate the specific aspergilli causing IFD. When GM is detected in serum or BAL fluid, but no culture results or other way of species identification are available, sampling should be repeated until a definitive diagnosis or ruling out of IA. In such cases of suspected IA, empiric treatment with AmB or a broad-spectrum triazole like isavuconazole, posaconazole or voriconazole may be initiated, and once a non-fumigatus Aspergillus spp. is identified, therapy may be adapted according to available MICs and local resistance patterns, if known. Therefore, a versatile mycology team should support the primarily caring clinical team in diagnosis and treatment, e.g., through an established ID consultation service, and awareness for IFD caused by rare fungal species needs to be created.

In conclusion, IFD is due to rare fungal species, and this may include *Aspergillus* spp. other than *A. fumigatus*, are complex to diagnose and treat. They have a high potential to be missed or detected late during the clinical evaluation and diagnostic work-up, while even skilled ID physicians may not be able to make ad-hoc decisions on treatment if diagnosed. Detailed, resource-orientated and species-specific guideline recommendations on diagnosis and clinical management for IA are upcoming by an initiative of the ECMM [126]. Larger studies are needed to determine differences in local epidemiology, risk groups and outcomes for each non-*fumigatus* entity.

## Data Availability

Not applicable.

## Supplementary Materials

The following supporting information can be downloaded at: https://www.mdpi.com/article/10.3390/jof9040500/s1, Table S1 Search Algorithms for PubMed Search.
