# Supplementary material for: Diagnosis and Treatment of Invasive Aspergillosis Caused by Non-fumigatus Aspergillus spp
Source: J Fungi (Basel). 2023 Apr 21;9(4):500. doi: 10.3390/jof9040500 (PMC10141595; doi:10.3390/jof9040500)
Supplement: Supplementary file 1 [file jof-09-00500-s001.zip › jof-2293713-supplementary.pdf]

**Supplementary File**

**Table S1. Search Algorithms for PubMed Search.**

| No. | Search algorithm |
|---|---|
| 1 | ("non-fumigatus"[MeSH Terms] OR "non-fumigatus"[All Fields] OR ("non-fumigatus"[All Fields] AND "diagnosis"[All Fields]) OR "non-fumigatus"[All Fields]) AND (invasive[All Fields] AND ("aspergillosis"[MeSH Terms] OR "aspergillosis"[All Fields])) |
| 2 | ("Aspergillus flavus"[MeSH Terms] OR " Aspergillus flavus"[All Fields] OR ("Aspergillus flavus"[All Fields] AND "diagnosis"[All Fields]) OR "Aspergillus flavus"[All Fields] AND "treatment"[All Fields] OR "Aspergillus flavus"[All Fields]) AND (invasive[All Fields] AND ("aspergillosis"[MeSH Terms] OR "aspergillosis"[All Fields])) |
| 3 | ("Aspergillus nidulans"[MeSH Terms] OR " Aspergillus nidulans"[All Fields] OR ("Aspergillus nidulans"[All Fields] AND "diagnosis"[All Fields]) OR "Aspergillus nidulans"[All Fields] AND "treatment"[All Fields] OR "Aspergillus nidulans"[All Fields]) AND (invasive[All Fields] AND ("aspergillosis"[MeSH Terms] OR "aspergillosis"[All Fields])) |
| 4 | ("Aspergillus niger"[MeSH Terms] OR " Aspergillus niger"[All Fields] OR ("Aspergillus niger"[All Fields] AND "diagnosis"[All Fields]) OR "Aspergillus niger"[All Fields] AND "treatment"[All Fields] OR "Aspergillus niger"[All Fields]) AND (invasive[All Fields] AND ("aspergillosis"[MeSH Terms] OR "aspergillosis"[All Fields])) |
| 5 | ("Aspergillus terreus"[MeSH Terms] OR " Aspergillus terreus"[All Fields] OR ("Aspergillus terreus"[All Fields] AND "diagnosis"[All Fields]) OR "Aspergillus terreus"[All Fields] AND "treatment"[All Fields] OR "Aspergillus terreus"[All Fields]) AND (invasive[All Fields] AND ("aspergillosis"[MeSH Terms] OR "aspergillosis"[All Fields])) |

Search performed between September 2022 and December 2022.
